# Use of rituximab as an induction therapy in anti-glomerular basement-membrane disease

**DOI:** 10.1186/s12882-018-1038-7

**Published:** 2018-09-20

**Authors:** M. Heitz, P. L. Carron, G. Clavarino, T. Jouve, N. Pinel, F. Guebre-Egziabher, L. Rostaing

**Affiliations:** 1 Service de Néphrologie, Hémodialyse, Aphérèses et Transplantation, CHU Grenoble-Alpes, Avenue Maquis du Grésivaudan, 38700 La Tronche, France; 2 Laboratoire d’Immunologie, CHU Grenoble-Alpes, La Tronche, France; 3 Laboratoire d’Anatomie Pathologique, CHU Grenoble-Alpes, La Tronche, France; 4 Université Grenoble-Alpes, Grenoble, France

**Keywords:** Rituximab, Goodpasture disease, Anti-glomerular basement-membrane disease, Induction therapy

## Abstract

**Background:**

Anti-glomerular basement-membrane (anti-GBM) disease (or Goodpasture disease) is characterized by severe kidney and lung involvement. Prognoses have improved with treatments that combine plasma exchange and immunosuppressive drugs. However, patients with severe renal involvement can have poor renal outcomes and cyclophosphamide can cause significant complications. Anti-GBM antibodies have a direct pathogenic effect on the disease: thus, therapeutics that can decrease their production, such as rituximab, could be a good alternative.

**Methods:**

The medical files of five patients that had received rituximab as a first-line therapy (instead of cyclophosphamide), plus plasma exchange and steroids, were reviewed. All patients had severe disease manifestations.

**Results:**

Four patients required dialysis at diagnosis and remained dialysis-dependent over the mean follow-up of 15 months. Three patients had pulmonary involvement, but recovered even though mechanical ventilation was required. Anti-GBM antibodies became rapidly undetectable in all patients. One infectious and two hematological complications were observed.

**Conclusions:**

We report the outcomes of five patients with Goodpasture disease and treated with rituximab as a first-line treatment. This strategy was effective at treating pulmonary manifestations and was associated with a good biological response with no major serious adverse events. However, renal outcomes were not significantly improved.

## Background

Anti-glomerular basement-membrane (anti-GBM) disease is an autoimmune disorder that is characterized by pulmonary hemorrhage and rapidly progressive glomerulonephritis, which usually manifests as acute renal failure. It is termed Goodpasture disease when both manifestations are simultaneously exhibited. This rare disease occurs in 1 person per million /year and generally evolves as a single acute phase, it relapses rarely [1]. Anti-GBM disease is caused by antibodies against non-collagenous domain-1 of the alpha-3 and alpha-5 chain in type IV collagen. The diagnosis is made by detecting circulating anti-GBM antibodies and a kidney biopsy provides a definitive diagnosis when there is renal involvement. Using light microscopy, focal proliferative or necrotizing crescentic glomerulonephritis is observed. The pathognomonic finding is linear deposition of immunoglobulin G (IgG) along the glomerular basement membrane seen by direct immunofluorescence [2].

Before the advent of plasmapheresis therapy, the mortality rate was ~ 90%. Today, patient survival at 1 year is 100% when creatinine level is < 5.6 mg/dL at presentation [3]. Reliable predictors for an adverse outcome include a serum-creatinine level of > 6.8 mg/dL, dialysis-dependence, and a percentage of crescentic glomeruli of > 50% at diagnosis [2–4]. The 2012 guidelines for Kidney Disease Improving Global Outcomes recommend initiating immunosuppression with cyclophosphamide plus corticosteroids and plasma exchange in all patients with anti-GBM glomerulonephritis, except for those that are dialysis-dependent at presentation and have 100% crescents on a biopsy and do not have a pulmonary hemorrhage [5]. However, this approach results in a significant proportion of patients remaining dialysis-dependent plus causing significant side-effects.

Anti-GBM antibodies can be pathogenic and their removal is associated with renal recovery [6]. The inhibition of their production through B-cell depletion could be of value. In renal vasculitis, e.g., ANCA-associated vasculitis, the use of the anti-CD20 monoclonal antibody, rituximab, has been reported to be efficacious in large randomized controlled studies [7, 8]. Herein, we report on the largest series of patients with Goodpasture syndrome, where the first-line immunosuppressive therapy was based on rituximab.

## Methods

We retrospectively reviewed the medical files of all patients with anti-GBM disease in our hospital between January 2013 to December 2016. Only five patients (1 male and 4 females) aged 18 to 75 years were treated during this period with Rituximab as first line therapy. Patients gave their verbal informed consent. Our local ethics committee (CPP Grenoble) ruled that no formal ethics approval is required in this particular case, i.e. routine patients ‘care. The patients’ characteristics are summarized in Table 1. At diagnosis, all five patients had serious clinical presentations with a median creatinine value of **>** 6.8 mg/dL (0.5–9). Four patients required dialysis within 7 days after diagnosis. Three patients (patient nos. 2, 3, 5) had an intra-alveolar hemorrhage and one of them (no. 3) required assisted ventilation. All patients were positive for anti-GBM antibodies. Two patients were double-positive with ANCA. Table 2 shows the clinical findings. A kidney biopsy was performed at diagnosis in three of the four patients that had renal impairment; the fourth patient declined a biopsy. Patients 2 and 5 had > 90% crescentic glomeruli; patient 1 had only 20%. Linear IgG staining without IgA was demonstrated on the glomerular basement membrane of all biopsies. Patient 3 did not have renal involvement. One patient was young (18-years-old) and rituximab was chosen because of the risk of infertility; the other patients had very poor renal prognosis, i.e. all were dialysis-dependent at diagnosis and cyclophosphamide has not demonstrated in this situation efficacy in helping recovery of kidney function. Finally, as these patients were old we thought that they were at high risk for cyclophosphamide-induced infectious complication. All patients received the same induction therapy with rituximab administered as four weekly pulses of 375 mg/m^2^ associated with daily plasma exchange (6 days/week) until the antibodies became undetectable (the replacement liquid was albumin, fresh-frozen plasma, or a combination). Pulses of methylprednisolone were given, followed by prednisone, which was gradually tapered. No patient received cyclophosphamide.

**Table 1:** Characteristics of the five patients

	At diagnosis
Age (median, ranges)	72 (17 to 96)
Gender (M/F)	1/4
Comorbidities	
Hypertension	2/5
Tobacco	2/5
Diabetes mellitus	0/5
Toxic exposure	0/5
Pulmonary symptoms	3/5
Mechanical ventilation required	1/5
Serum creatinine μmol/L (median, ranges)	605 (47 to 800)
Dialysis required at diagnosis	4/5
Proteinuria g/L (median, ranges)	4 (0.2 to 6)
Biological characteristics	
Hemoglobin g/L (mean, ranges)	95 (76 to 116)
Anti-GBM titer of dilution (IIF) (mean, ranges)	97 (5 to 640)
ANCA +	2/5
Crescents > 50% on biopsy	2/3
Treatment	
Number of plasma-exchange sessions, mean (range)	16 (9 to 23)
Corticosteroids	5/5
Rituximab as a first-line treatment	5/5

*Abbreviations: F* female, *M* male, *IIF* indirect immunofluorescence, *ANCA* anti-neutrophil-cytoplasmic antibody, *anti-GBM* anti glomerular-basement-membrane

**Table 2:** Patients characteristics and treatment summary

Patient	Initial clinical presentation	Creatinine at diagnosis	Iinitial dialysis	Crescents	Initial	ANCA	Number of plasma exchange sessions	Corticosteroids	Rituximab	Outcome	Complications	Follow up duration	Evolution
		(μmol/L)	within 7 days after diagnosis	on biopsy	anti-GBM titer (IIF)							(months)	
Patient 1	general weakness, AKI	706	yes	20%	1/10	negative	9	120 mg ×3 + 1 mg/kg	375 mg/m2 ×4	ESRD	no	39	Transplantation
Patient 2	AKI with diarrhea, dyspnea and desaturation, alveolar hemorrhage	800	yes	90%	1/80	positive, MPO	23	1 mg/Kg	375 mg/m2 ×4	Pulmonary recovery, ESRD	no	23	Hemodialysis
Patient 3	hemoptysis, respiratory distress with mechanical ventilation, AKI in intensive care	47	no	NA	1/5	positive, non specific	10	1 mg/kg	375 mg/m2 ×4	Pulmonary recovery	candida colonization	4	Creatinine 48 μmol/L
Patient 4	rapidly progressing glomerulonephritis, hematuria	273	yes	NA	1/640	negative	21	500 mg × 4 + 1 mg/kg	375 mg/m2 ×4	ESRD	Esophageal candidiasis + temporary	9	Peritoneal dialysis
											thrombocytopenia		
Patient 5	hemoptysis,AKI	605	yes	100%	1/200	negative	20	500 mg × 3+ 1 mg/kg	375 mg/m2 ×4	Pulmonary recovery, ESRD	no	14	Transplantation

*Abbreviations*: *ESRD* end stage renal disease, *F* female, *M* male, *AKI* acute kidney injury, *IIF* indirect immunofluorescence, *ANCA* anti-neutrophile-cytoplasm antibody, *anti-GBM* anti glomerular-basement-membrane antibody, *NA* data not available

All patients recovered rapidly from the lung injury, even the patient that required mechanical ventilation (no. 3). However, there was no renal recovery within a median follow-up of 14 months (range 4–39) in the four patients that were dialysis-dependent at presentation. Antibodies became undetectable in all five patients by a median of 19 days (range 2–26) after the first plasma exchange and by a median of 19 days (range 5–30) after the first perfusion of rituximab. Antibodies then remained undetectable during the follow-up period. One infectious and two hematologic (initial thrombocytopenia, leucopenia at 6 months) complications, within the same patient, were observed. During the follow-up, two patients received a kidney allograft. No death or relapse occurred in any of the five patients.

## Discussion

We report the outcomes of five patients that had Goodpasture disease and whose initial induction treatment was based on rituximab (instead of cyclophosphamide), plus prednisone and plasma exchange. Although pulmonary injury was reduced and the biological responses were good with no relapses, the renal outcomes were not improved.

Anti-GBM antibody-mediated disease is a fulminant disorder that has a poor prognosis and a mortality rate of up to 90% without treatment. However, despite intensive treatment, many patients (60%) remain dialysis-dependent [4] and have adverse complications from immunosuppressive treatments [3]. Since the discovery of a direct pathogenic effect of anti-GBM antibodies, therapies have aimed to decrease the production or increase the clearance of these antibodies. Thus, rituximab, an anti-CD20 monoclonal antibody, could be a good alternative to cyclophosphamide.

In the literature, the first use of rituximab for anti-GBM disease was reported in 2002 by Arzoo et al. for a patient with a pulmonary hemorrhage and that was refractory to conventional treatments [9] (Table 3A). Since this case, several small studies have been published, which suggest a good pulmonary response and good overall survival of patients, although renal outcomes were not significantly modified [10–12].

**Table 3:** Case series when using rituximab therapy in the setting of Goopasture syndrome as a second-line (A) or first-line (B) therapy

Article	Gender	Age	Clinical findings	Renal injury	Pulmonary symptoms	Creatinine μmol/L (GFR ml/min/1.73m^2^)	Anti-MBG at diagnosis or at initiation of rituximab	Previous treatment	Rituximab indication	Treatment associated with rituximab	Rituximab	Renal outcome	Pulmonary symptom	Anti-MBG U/ml
A. RITUXIMAB AS A SECOND-LINE THERAPY														
Arzoo et al.	F	73	hypoxia, haemoptysis, mechanical ventilation, haematuria	no	yes	ND	51(ELISA)	CYC,PE, CTC	relapse at one year, 2nd treatment failure	CYC PO, corticosteroids	6 times weekly 375 mg/m^2^	recovery	no	undetectable
Shah et al.	M	54	severe renal failure, anuria, hypertension	yes	yes	1874 (dialysis dependent)	680 (U/ml)	CYC IV (500 mg) one dose, PE = 50, corticosteroids	haematological complication of CYC after one dose (on day 5)	PE = 50, corticosteroids	4 times weekly 375 mg/m^2^	dialysis dependent	no	undetectable
	M	64	nasal obstruction, nausea, weight loss, AKI	yes	ND	536 (dialysis dependent)	49 (ELISA)	CYC PO (50 mg/d) for 7 days, PE, corticosteroids	haematological complication of CYC after 7 days	PE, corticosteroids	4 times weekly 375 mg/m^2^	dialysis independent after 4th rituximab, creatinine 260 μmol/L	ND	undetectable
Syeda et al.	F	68	renal failure, TTP	yes	no	994(dialysis dependent)	1/160 (IIF)	CYC (2 mg/kg/d) for 5 days, PE, corticosteroids	haematological complication of CYC after 5 days	PE, corticosteroids	D28, 4 times weekly 375 mg/m^2^	dialysis dependent	no	undetectable
Touzot et al.	F	21	renal failure, pulmonary haemorrhage	yes	yes	GFR 27	0 (ELISA)	CYC IV (1000 mg) PE = 13, corticosteroids	severity of disease	corticosteroids	4 times weekly 375 mg/m^2^	GFR 35	no	undetectable
	F	46	renal failure	yes	no	dialysis dependent	1/200 (IIF)	CYC IV (1000 mg) PE = 15, corticosteroids	severity of disease, PE dependency	corticosteroids	4 times weekly 375 mg/m^2^	dialysis dependent	no	undetectable
	F	16	renal failure, haemodynamic instability	yes	no	GFR 105	0 (ELISA)	CYC IV (2000 mg) PE = 1,1 corticosteroids	severity of disease (ECMO)	corticosteroids, MMF 720 mg/d	4 times weekly 375 mg/m^2^	GFR 103	no	undetectable
	M	65	ND	yes	ND	GFR 17	1/40 (IIF)	CYC IV (2000 mg) PE = 9, corticosteroids	persistent anti-GBM antibodies	corticosteroids, MMF 720 mg/d	4 weekly 375 mg/m2	GFR 25	no	undetectable
	F	19	ND	yes	ND	GFR 29	25(ELISA)	CYC IV (1400 mg) PE = 25, corticosteroids	PE dependency	CYC IV (500 mg) PE = 10 corticosteroids	4 times weekly 375 mg/m^2^	GFR 96	no	undetectable
	M	22	ND	no	yes	GFR 126	19(ELISA)	CYC IV (4500 mg) PE = 0, corticosteroids	relapse at 3 years, alternative therapy	corticosteroids	4 times weekly 375 mg/m^2^	GFR 108	no	undetectable
	F	17	ND	yes	yes	GFR 53	8 (ELISA)	CYC IV (3000 mg),PE = 0, Corticosteroids	Relapse at 6 months after decrease of corticosteroids	PE = 6,CYC = 60 0 mg ×3, Prednisone	4 times weekly 375 mg/m^2^	Dialysis dependent	no	undetectable
	F	21	ND	yes	no	GFR 46	40(ELISA)	CYC IV (900 mg) PE = 0, corticosteroids	severity of disease	CYC IV (900 mg) PE = 19 corticosteroids	4 times weekly 375 mg/m^2^	GFR 74	no	undetectable
Sauter et al.	M	29	renal failure, pulmonary haemorrhage	yes	yes	140	43	CYC, PE =17, corticosteroids	relapse at 18 months after transplantation	PE, MMF 3000 mg/d then replaced by CYC PO, corticosteroids	3 weekly 375 mg/m2	dialysis dependent, graft lost	no	undetectable
Bandak et al.	M	24	Haemoptysis haematuria, AKI	yes	no	379	161	CYC PO (150 mg/d) 6 months, PE = 17, corticosteroids	treatment failure	CYC+ corticosteroids	one dose, 1000 mg	creatinine 181 μmol/L	no	undetectable
B. RITUXIMAB USED AS A FIRST-LINE THERAPY														
Wechsler et al.	M	55	Haematuria, AKI in HIV, diabetic patient with septic hip arthritis	yes	no	310	8,6	no	infectious risk	MMF 1000 mgx2/d, corticosteroids IgIV	4 times weekly 375 mg/m^2^	creatinine 106 μmol/L	no	undetectable
Shah et al.	M	17	nausea, weakness, haemoptysis, weight loss	yes	yes	272	131	no	fertility	PE = 17, corticosteroids	2 times weekly 375 mg/m^2^	creatinine 99 μmol/L	no	undetectable
Narayanan et al.	M	21	Oliguria, weakness, oedema, haemoptysis	yes	yes	1126	191	no	fertility	PE = 5, corticosteroids	2 doses, two weeks apart	dialysis dependent	no	undetectable

*Abbreviations: ESRD* end-stage renal disease, *F* female, *M* male, *AKI* acute kidney injury, *IIF* indirect immunofluorescence, *ANCA* anti-neutrophil-cytoplasm antibody, *anti-GBM* anti-glomerular basement membrane antibody, *MMF* mycophenolate mofetil, *PE* plasma exchange, *GFR* glomerular-filtration rate, *CYC* cyclophosphamide, *IgIV* immunoglobulin intravenous, *PO* per os, IV intravenous, *ECMO* extracorporeal membrane oxygenation

Currently, an initial induction treatment with rituximab, instead of cyclophosphamide, has been described in only three case reports (Table 3B). Wechsler et al. reported on a case with renal involvement (baseline plasma creatinine: 3.5 mg/dL, tubular-cell necrosis, and only one crescent on a biopsy) but no pulmonary symptoms. The patient had an HIV infection (human immunodeficiency virus), mellitus diabetes, and septic hip arthritis. He was treated with rituximab combined with prednisone, IV immunoglobulin, and mycophenolate mofetil to avoid infection. Evolution was good: at one month after initiation of therapy, renal function was improved (creatinine 1.1 mg/dL and antibodies were undetectable). This patient presented with only one complication, *Candida* esophagitis. In this case, a poor prognosis was not identified at diagnosis**,** thus, it is possible that the resolution of tubular-cell necrosis contributed to the renal recovery [13].

The second case report, by Shah et al., was on an 18-year-old patient that was treated with plasma exchange, prednisone, plus rituximab to preserve fertility because of his young age. He presented with major pulmonary injury and moderate renal impairment (80% of glomeruli were involved as shown by cellular crescents in a kidney biopsy and baseline plasma-creatinine of 3 mg/dL). Antibodies became undetectable at 20 days after initiating rituximab. No complications were reported and, after a follow-up of 33 months, he had renal (plasma creatinine of 1.1 mg/dL) and pulmonary recovery [14].

The third case report, reported by Narayanan et al., describes a young man with pulmonary and acute kidney injury that required dialysis (creatinine at diagnosis 12.8 mg/dL and 100% crescents in a kidney biopsy). He received plasma exchanges, corticosteroids and rituximab. After 4 months of follow-up, he had completely recovered from the pulmonary hemorrhage, the antibodies had become undetectable, but he remained dialysis dependent [15].

Only 16 other cases have been reported on anti-GBM disease treated with rituximab as a second-line therapy [9, 11, 13–16] (see Table 3A). In most cases, the treatment strategies were heterogeneous and cyclophosphamide was used as the first-line therapy. Rituximab was initiated primarily as a rescue therapy because the initial conventional treatment had failed or there were serious adverse events. Among these published cases, four patients had very poor renal prognoses (creatinine > 6.8 mg/dL or were dialyzed at presentation) of whom three remained dialysis-dependent despite treatment [10, 11, 14]. Among 12 patients that were not dialysis-dependent at baseline, two patients did not recover renal function and progressed to end-stage renal disease [10, 12]. Pulmonary symptoms were identified in six patients and all recovered [9, 10, 12, 14]. Among the 16 patients, rituximab was administered for a disease relapse that was limited to the lungs in two patients; these relapses occurred at 1 and 3 years after the initial therapy. These two patients were successfully treated and had no complications [9, 10]. Renal relapse developed in two other patients, this occurred at 6 months after the initial treatment, with cortico-dependency [10], in one case and at 36 months after transplantation [12] in the second case. Both these patients progressed to end-stage renal disease despite the use of rituximab.

Because anti-GBM antibodies are considered pathogenic their titers were monitored in most published studies. Cui et al. reported that anti-GBM antibodies remained positive for ~ 29 days with conventional treatment that combined plasma exchange, cyclophosphamide, and corticosteroids [1]. In most cases, a good biological response to rituximab was reported but the number of plasma exchanges required varied from 10 to 50 sessions. Similar to these observations, we found a clearance in anti-GBM antibodies within a median time of 19 days (range 2–26) after rituximab infusion.

Anti-GBM removal is usually achieved after a median time of 19 days of plasma exchange, but immunoadsorption can also be efficacious. Bisenbach et al., in 2014, described ten patients treated with immunoadsorption plus prednisone and cyclophosphamide. This protocol enabled rapid removal of anti-GBM antibodies (undetectable within 2–9 immunoadsorption sessions, i.e. < 1 month) and led to rapid clinical remediation of the pulmonary hemorrhage and improved renal function. Three of six patients that were dialysis-dependent remained free of dialysis for 64, 23, and 9 months. To our knowledge, there are no data on the efficacy and safety of immunoadsorption combined with rituximab to treat anti-GBM disease [17].

The cases reported in the literature have several limitations. The indications, the times to initiation of rituximab treatment, durations of treatment and total doses of rituximab, and the range of combined treatment strategies varied between patients. However, the reported outcomes uniformly suggest a good pulmonary response although renal outcomes were not significantly modified. The survival of patients was excellent and almost no complications occurred. This agrees with our findings.

The strength of our study is that all of our patients received homogeneous similar treatments. However, the retrospective nature of our study, the limited number of patients, and the lack of a comparator control group with another strategy to deplete anti-GBM antibodies, prohibits us from forming strong conclusions. Our patients had poor prognostic indicators at presentation, including high plasma creatinine and high percentages of cellular crescents on kidney biopsies.

## Conclusions

This is the first report to include five patients that received rituximab as part of a first-line induction therapy for anti-GBM disease. Our findings suggest that rituximab effectively induced complete resolution of pulmonary hemorrhage. It was associated with a good biological response with no major life-threatening adverse events. However, renal outcome was not significantly improved for patients that were dialysis-dependent at presentation. Whether other strategies that combine B-cell depletion and the removal of antibodies with immunoadsorption can be more effective and needs investigation.

## Abbreviations

Anti-GBM: Anti-glomerular basement-membrane autoantibody
